# The Effects of BITs on Contract Execution in Developing Countries: Some Implications for the COVID-19 Crisis

**DOI:** 10.3389/fpubh.2022.839030

**Published:** 2022-03-31

**Authors:** Zhihan Yu, Chao Zhou, Qin Wang, Ke Hu, Ke Su, Mengqi Zhang

**Affiliations:** ^1^School of Smart Finance and Economics, Chongqing City Management College, Chongqing, China; ^2^School of Business English, Sichuan International Studies University, Chongqing, China; ^3^Postdoctoral Mobile Station, Southwest University of Political Science & Law, Chongqing, China; ^4^School of Economics and Management, Heilongjiang Bayi Agricultural University, Daqing, China; ^5^Chongqing College of Electronic Engineering, Business School, Chongqing, China

**Keywords:** post-COVID-19 era, BITs, developing countries, contract execution, panel data

## Abstract

Bilateral investment agreements are bilateral treaties between capital exporting countries and host countries, which is specially used to protect international investment, and the contract execution is directly related to the daily operation of multinational enterprises. Based on the panel data of 73 developing countries from 2005 to 2019, this paper examines the improvement effect of BITs on the contract execution of host countries. The study found that both the overall bilateral investment agreements and the bilateral investment agreements in force can significantly improve the contract execution of the host country. Due to the differences between the civil law system and the common law system in many aspects, such as the source of evidence and trial mode, the effect of BITs on the improvement of contract execution in host countries of the common law system is more prominent. In terms of specific impact, the improvement effect of BITs on time is significantly better than cost. The core conclusion is still valid after changing the estimation method and eliminating abnormal samples.

## Introduction

Developed countries began to sign the bilateral investment treaties (BITs) with many developing countries in 1959 to protect their foreign direct investments (FDI) and other economic interests. With the continuous development of economic globalization and international trade, the signing parties of BITs have expanded from the original developed and developing countries. The number of treaties among developing countries has also increased, but parties are still developed and developing countries (1). According to the United Nations Conference on Trade and Development (UNCTAD), (2), 2,957 global BITs had been reached by 2016. For capital-exporting countries, the main purpose of BITs is to secure domestic foreign investment and strive for better market entity treatment for multinational enterprises. BITs can attract more FDI (3). With closer economic and trade relations between countries, the role of BITs is no longer limited in the flowing of investment from capital-exporting countries to developing countries and has a far-reaching impact on the political and economic system of the host country. For example, Pinto and Zhu (4) studied the relationship between FDI and corruption in host countries. They found that FDI can inhibit corruption in host countries with a low degree of democracy.

For a long time, the academic research on BITs has mainly focused on two aspects: the legal effect of BITs and the improvement of their legal provisions (5). The other is to analyse the facilitation effect of BITs on FDI (6–8). Especially under the background of the rapid increase of BITs and the increasingly frequent flow of FDI worldwide, scholars pay more attention to BITs in promoting FDI flow. In addition, with the continuous growth of FDI inflows in developing countries, some scholars have also observed its spillover effect on the institutional quality of host countries (9–11).

The existing research has carried out some good exploration on the relationship between international business activities and the institutional quality of host countries and made fruitful achievements. However, there are still deficiencies in the following two aspects. Firstly, the research on BITs at home and abroad mainly focuses on their impact on FDI, ignoring the facilitation effects on improving the institutional quality of developing countries. Secondly, when analyzing the impact of international economic and trade activities on the institutional quality of host countries, papers use macroeconomic indicators, which are weakly related to the daily business activities of enterprises, such as the economic management index and authorities' governance quality. Although these indicators can comprehensively reflect a country or region's institutional quality, they mainly focus on the macro level and pay less attention to the micro-environment closely related to enterprises.

Because of the shortcomings of the existing research, this paper focuses on 73 developing countries as the study subject and analyses the impact of BITs on the contract execution of the host countries. Developing countries have less complete and more arbitrary law systems than developed countries, so many multinational enterprises face greater external risks when investing abroad. Although a customary international law restricts the behavior of capital-exporting countries and host countries, the protection that this law can offer to foreign investors is very little for the lack of recognized norms and dispute settlement mechanisms (12). Capital-exporting countries often sign BITs with the host country to help domestic enterprises avoid external institutional risks and protect their economic interests. Although the specific contents of BITs vary from country to country. As one of the most important international institutions to protect FDI, BITs have covered more than 180 countries and regions by 2016 (2). According to the definition of the World Bank, contract execution mainly measures the time and cost of resolving commercial disputes through local first instance courts, as well as the quality index of judicial procedures, and evaluates whether each economy has adopted a series of good practices to improve the quality and efficiency of the judicial system. The project covered over 190 economies in 2018, increasing from 133 economies in 2002. It has become one of the authoritative data for studying a country's business environment or region and has been adopted by several papers (13, 14).

Based on the above analysis, the contribution of this paper is mainly reflected in the following two aspects: First is to expand the research scope of BITs from FDI and other business activities to the institutional quality of the host country. Although the main function of BITs is to promote the effective flows of FDI, the existing research ignores its spillover effect on the institutional quality of the host country under the situation of the increasingly complex international economy and trade. The second is to deepen the existing research and take the contract execution closely related to the daily business activities of enterprises. Presently, the research on BITs and institutional quality mainly start from the broader institutional systems, such as political and economic systems. Due to the low attention of multinational enterprises to these systems, taking these systems as the study subject would fail to reflect the impact of BITs on the quality of micro institutions of the host country. Based on the overall analysis, this paper also examines the impact of BITs on specific aspects of contract execution in order to provide more practical and effective policy suggestions for the host country to improve its business environment.

## Mechanism Analysis of BITs to Improve Contract Execution in Host Countries

The contract execution is the central content of contract system, which is not only directly related to the economic interests of bilateral commercial subjects, but also closely related to the economic development of a country. BITs are an important part of the international investment law. Based on the basic framework and combined with the specific conditions of each country, a lot of work has been carried out in terms of investment definition, access and treatment of foreign investment and dispute settlement mechanism. Combined with researches of Kerner (15) and Falvey and McGregor (3), this paper holds that the influence of BITs on the contract execution in host countries is embodied in four aspects, such as strengthening the institutional foundation of domestic law of host countries, improving procedural legal provisions, providing a reference and specific path for dispute resolution, and ensuring or reinforcing the effectiveness of implementation.

### BITs Further Strengthen the Institutional Foundation of Host Countries' Domestic Law

Different from that of developed countries, the legal foundation of developing countries is relatively weak, and some concepts are not clearly defined, which further restricts the application and execution of laws. The clear definition of concepts is the basic requirement of contract execution, which delineates the particular scope of law enforcement. In order to maximize the protection of their own investment, capital-exporting countries conduct consultations over and over again with host countries on the specific concepts involved in BITs, so as to create favorable conditions for resolving potential investment disputes. Taking the BITs signed by China and Kazakhstan in 1992 as an example. The concepts of “investment” and “investor” were defined in the first paragraph of Article 1 and listed key forms. In addition, although the number of BITs signed among developing countries grows rapidly, those signed between developed countries and developing countries are still dominant (1). Developing countries have worked out BITs through constant consultation with developed countries with better legal systems to bridge gaps in relevant fields of domestic laws. More importantly, the gradual improvement of the legal basis related to BITs provides specific reference for host countries to clarify concepts in other fields, promoting the overall improvement of the efficiency of contract execution in host countries.

### BITs Further Improve the Procedural Legal Provisions of Host Countries' Domestic Law

Economic behavior is established on the institutional environment and subject to the constraints and norms stipulated by law, so the pursuit of profit of capital promotes the continuous improvement of procedural law and arbitration mechanism, directly or indirectly. In the handling and enforcement of foreign-related investment disputes or international investment disputes, the arbitral tribunal shall examine whether host countries have fulfilled their obligations or abused their power under the investment agreement in order to ensure the rationality, fairness and accuracy of the commercial award. Since the arbitral tribunal has certain “discretionary space”, it is deficient in terms of the consistency and transparency of the award. Additionally, to maximize the protection of their own interests, transnational enterprises often sponsor business associations and legal organizations in host countries, and then influence the ruling results of cases as a third party. Due to the combined action of the above multiple factors, the benefits of the award results tend to be tilted to transnational enterprises. In purpose of reducing their passivity in the international arbitration, host governments will clarify the specific requirements of relevant regulations in BITs. Compared with the settlement of domestic investment disputes, the settlement of investment disputes involving transnational factors is more difficult in terms of language and space distance. The scope of constitutive elements is broader and fuzzy due to the heterogeneity of the two countries' systems, the differences in the interpretation of regulations and agreements, and the definition of specific applicable rules of treaties. In this context, the relatively complex legal process and system of international dispute settlement supplement the domestic procedural law of host countries. The abuse of process such as procedural obstruction and procedural delay exposed by the arbitration system also provides many cases for the settlement of similar cases in China. Under the dual track system of arbitration supervision, clear, reasonable and comprehensive BITs make the supervision and trial carried out by the judicial authorities of host countries operable, which means the procedural review of foreign awards has spillover effects on the domestic procedural law.

### BITs Provide a Reference and Specific Path for the Settlement of Domestic Disputes in Host Countries

BITs provide the means and procedures not only for the settlement of disputes arising from the interpretation and implementation of treaties among contracting states, but also for that of disputes arising from investment between foreign investors and governments of host countries, especially most agreements resolve disputes through International Center for Settlement of Investment Disputes (ICSID). These measures provide a strong guarantee for the proper settlement of investment disputes. As the construction and operation of international dispute mechanism inevitably involve the public law factors such as politics and sovereign, the integration of government resources in the industrial ecology, such as industrial policy and institutional system, plays a leading role in the normative system, and the diversified settlement and arbitration mechanism of international trade and investment disputes fill the space for the creation of rules, which constructs a legal framework for the settlement of domestic disputes in host countries. Based on the particularity of economic laws of contracting parties in terms of scope of application and meaning, in order to reduce the difficulty of adjudicating disputes and improve the efficiency and quality of dispute resolution, host countries may make effective reference to the legal situation of capital-exporting countries through BITs. Furthermore, on the basis of ensuring the inclusiveness and adaptability of the system, similarities and differences between the legal cultures and functions of the two countries are explored to further promote the reform and innovation of the legal system. In addition, host countries expand the extension of their laws through comparative study of bilateral laws, which increases the reference of domestic legal profession to actual cases and provides a useful reference for the settlement of relevant disputes in the context of international market.

### BITs Can Ensure or Reinforce the Effectiveness of Implementation of Domestic Law

International dispute settlement mechanism and domestic judicial mechanism are involved in the construction of a predictable and innovative business environment, which means maintaining international investment order requires a balanced consideration of resources at home and abroad. Investment arbitration and specific contract execution are related to the balance between the protection of investors' interests and the regulation rights of host countries, as well as the value judgment of the investment laws and control policies of host countries. The ruling focuses on promoting investment relations and maintaining investment order through the balance of interests. Regional economy is established on organizational rules and entities, and the force of law is an important premise to ensure that practice does not exceed reasonable expectations. In the context of international dispute settlement where a temporary organization tasked with arbitrating a dispute between two parties applies rules or principles of international law to evaluate the law or public policy of host governments, host countries often take measures such as expanding legal sources, enhancing legal effectiveness and adaptability to reduce the risk of losing a lawsuit and the transaction cost of the system, so as to get rid of the situation that the legitimate regulation power is controlled by the arbitral tribunal. The development of international investment dispute settlement methods promotes the improvement of host countries' overall business environment and institutional system. Host countries indirectly strengthen the effectiveness of domestic laws while adjusting their own foreign investment laws and public policies. Compared with international law, domestic law has more enforceable effectiveness and more precise adjustment subjects. When investment disputes are transferred from international to domestic, the effective time and conditions of law are more disposable. Although the governance structure continues to change and market demands are becoming more diversified, the rule of law is still the main line in building a business environment. The decision that state parties explicitly regard the investment arbitration award as the scope of domestic law means that the investment treaty arbitration also belongs to the scope of domestic law, rather than the scope of public international law, which reflects that domestic law and international law have a certain crossover and the scope of the boundary presents a narrowing trend. With the increasingly introverted international obligations, host countries are subject to regulation by adopting a specific legal framework within their own legal system, granting specific rights, and avoiding specific acts.

## Empirical Models, Variables, and Data Sources

Based on the research of Haddad and Hornuf (14) and Long et al. (11), this paper analyses the effect of BITs on the improvement of the contract execution in developing countries by using the data of the business environment project of the UNCTAD and the World Bank. The specific model is as follows:


(1) $$\begin{array}{l} \text{ln}contract_{it}=c+\beta_{1}\ln treaties_{it}+\beta_{2}lnpgdp_{it}+\beta_{3}\ln trade_{it}\\ \;+\beta_{4}resources_{it}+\beta_{5}control_{it}+\varepsilon_{it} \end{array}$$

In the above formula, _*i*_ and _*t*_ represent the country and year respectively. _*c*_ represents the constant term. _β_ represents the influence coefficient of each variable. _*contract*_ refers to the contract execution. _*treaties*_ represents the number of BITs officially entered into force in developing countries. _*pgdp*_ represents the per capita gross domestic product (GDP). _*trade*_ is the trade openness, _*resources*_ is the resource abundance. _*control*_ is the level of corruption control. _ε_*it*__ is a random error term.

### Dependent Variable

It is the contract execution in developing countries. The world bank's business environment project has published contract execution data of various countries and regions since 2005. With the continuous improvement of the research system, the world bank has added filing and service, trial and judgment and other indicators on the basis of procedures, time and cost. Among the indicators of contract execution, time and cost are directly related to the economic benefits of enterprises, so multinational enterprises pay the most attention to these two indicators. On the other hand, in order to ensure the continuity of research data and investigate the possible heterogeneous impact of BITs on contract execution in developing countries after the 2008 financial crisis, this paper will mainly use time and cost to construct contract execution and carry out further research.

### Main Variable of Interest

It is the number of BITs. At present, the measurement methods of BITs are generally divided into two categories. One is to use the number of signed BITs. The other is that BITs are set as dummy variables. The main reason for this difference lies in the different research categories. For example, Zeng and Lu (16) mainly focus on the impact of treaties on FDI and international trade.

Meanwhile, Mazumder (17), and Sirr et al. (8) study the impact of BITs on attracting FDI and leaders' ruling time. Mazumder (17) takes many developing countries or regions as the research subject, and several papers take a single country as the research subject. Although the number of BITs signed between developing countries continues to grow, the treaties signed between developed and developing countries are still dominant in terms of overall quantity and impact. At the same time, the stock of foreign investment in developed countries is large, and the level of their business environment is high. In addition, for reasons like institutional coordination and legal procedures, many BITs will not enter into force in the current period. Although treaties that have not entered into force do not have substantive effectiveness, capital-exporting countries can also indirectly affect the business environment of the host country.

Improving the business environment in developing countries is a long-term process, and the data on the stock of BITs reflects its sustainable impact. Moreover, many treaties reflect the greater pressure of capital-exporting countries on improving the business environment of the host country and provide a valuable opportunity for the host country to understand and learn from other countries the system of the business environment and reform experience. Based on the research subject of this paper and the effects of BITs, this paper will follow the practice of scholars, such as Sirr et al. (8). It also takes the cumulative number of treaties signed with developed countries as the proxy variable. While mainly examining the overall number of BITs (including those that have entered into force and those that have not), this paper will also focus on whether the treaties in force have the same effect. In addition, the newly signed BITs are only a supplement and improvement to the original treaty, so this paper mainly focuses on the year when the first treaty was signed.

### Control Variables

The contract execution of developing countries is not only affected by BITs but also closely related to economic development, international trade, the abundance of natural resources, corruption control. Therefore, the above variables are added to the model as control indicators.

### Economic Development

With the continuous improvement of economic development, developing countries have a more urgent need for an efficient legal system, but also have a more sufficient material foundation support. Referring to Du and Sun (18), this paper chooses the per capita GDP of developing countries to reflect the country's economic development.

### International Trade

International trade plays an important role in a country's economic development and promotes cultural and personnel exchanges, which is with substantial impact on institutional system of developing countries (19). This paper selects the proportion of total import and export trade in the total GDP of each country as an indicator for measuring international trade.

### The Abundance of Natural Resources

According to the “resource curse hypothesis”, the relatively abundant natural resources create favorable conditions for a country's market rent-seeking behavior, thus hindering institutional quality improvement. Referring to Hamilton (20), this paper adopts the proportion of the total rent of natural resources in a country's GDP to measure the abundance of natural resources.

### Level of Corruption Control

Considering the important role of government in the construction of institutional system, government governance will directly affect the business environment in developing countries (21). This paper adopts corruption control to measure the political environment.

As the coverage of the world bank's business environment project has been expanding, the early data of some countries and regions are vacant. Based on the availability of data, this paper finally selects 73 developing countries and the research time range is from 2005 to 2019. According to the statistics of the United Nations Conference on Trade and Development, as of 2019, 73 developing countries have signed 2,788 BITs, and the number of BITs that have entered into force is 2003. It should be noted that both indicators account for more than 90% of the total number, indicating that the sample in this paper is well representative. The descriptive statistical results of the main variables are shown in Table 1.

**Table 1:** Descriptive statistic.

**Variable**	**Variable name**	**Observations**	**Average**	**Std. dev**.	**Minimum**	**Maximum**	**Data sources**
*be*	contract execution	1,095	62.79	11.68	33.60	88.41	World Bank Doing Business
*treaties*	bilateral investment treaty	1,095	33.85	24.20	3	109	United Nations Conference on Trade and Development
*pgdp*	per capita GDP	1,095	6272.71	7876.46	292	55,494.95	World Bank World Development Indicators
*open*	International trade	1,095	86.30	34.45	22.49	208.31	World Bank World Development Indicators
*resources*	abundance of natural resources	1,095	9.42	12.24	0.01	61.95	World Bank World Development Indicators
*control*	level of corruption control	1,095	42.84	12.40	19.54	81.65	World Bank World Governance Indicators

Based on the data availability of business environment and other variables, the final research scope of this paper is determined to be 73 developing countries from 2005 to 2019, respectively. For the sake of space, we do not list 73 developing countries in detail, but they are available upon request. By 2016, 73 developing countries had signed 2,598 BITs in total, accounting for about 88% of the total 2,957 BITs globally, of which 1,923 had entered into force. The descriptive statistical results of the main variables are shown in Table 1.

## Empirical Results

The empirical test of this paper is mainly carried out from three aspects: first, it tests the core conclusion of this paper, that is, whether BITs improve the contract execution of developing countries. The second is to discuss the potential differential impact of BITs between civil law countries and common law countries. The third is to further investigate the possible heterogeneity effect of BITs on contract execution in developing countries after the financial crisis. On this basis, this paper will test the robustness from two aspects: research methods and abnormal samples, so as to ensure the credibility of the core conclusions.

### Baseline Regressions

According to Eq. (1), the panel fixed-effects method, based on robust standard errors, is used to analyse the impact of BITs on the contract execution of 73 developing countries. According to the results of the Hausman test, this paper uses the fixed-effects estimation to carry out regression analysis. The results are shown in Table 2. It should be noted that although this paper may face endogeneity problems caused by omitted variables, the fixed-effects model of panel data can eliminate the bias caused by unobservable factors, thus effectively avoiding this problem (22).

**Table 2:** Estimation results (entire sample, 2005–2016).

	**BITs in general**		**BITs in force**	
	**(1)**	**(2)**	**(3)**	**(4)**
*ln treaties*	0.074^***^ (4.52)	0.040^***^ (2.76)	0.074^***^ (5.19)	0.048^***^ (3.02)
*ln pgdp*		−0.006 (-0.98)		−0.008 (-1.18)
*lnopen*		0.027^**^ (2.55)		0.027^**^ (2.53)
*resources*		0.048^**^ (2.42)		0.056^**^ (2.43)
*control*		−0.008^**^ (-2.16)		−0.007^**^ (-2.01)
constant	2.757^***^ (13.61)	1.494^***^ (5.14)	3.065^***^ (13.81)	1.620^***^ (5.61)
R^2^	0.2455	0.2236	0.2376	0.2622
Observations	1,095	1,095	1,095	1,095

***, **, * *means significant at 1, 5, and 10% levels respectively. The values reported in parentheses are t-values*.

As can be seen from Columns (1) and (2), the overall coefficients of BITs are positive and significant at the 1% level. This result shows that the increase in the total volume of BITs significantly improves the contract execution in developing countries. According to the results of columns (3) and (4), the coefficients of BITs in force are also significantly positive, which indicates that they also have a significant positive impact on the contract execution of developing countries. It is worth noting that the overall coefficient of BITs is slightly smaller than that of BITs that have entered into force. This result shows that the effective BITs have a more prominent improvement effect on the contract implementation of developing countries.

In terms of control variables, the economic development has not passed the significance test, which shows that it has no substantive impact on the contract execution of developing countries. The coefficient of foreign trade is significantly positive and has passed the significance test at the level of 5%, indicating that the sustainable development of foreign trade is helpful to promote the contract execution. Similar to the results of foreign trade, the improvement of corruption control in developing countries also helps to promote the contract execution. Consistent with the expectation of this paper, the abundance of natural resources has a significant restrictive effect on the contract execution as an important part of the institutional quality of developing countries.

### Results Based on Civil Law System and Common Law System

Due to the differences in social system, history and culture, the current legal systems of various countries in the world can be roughly divided into civil law system and common law system. There are significant differences between civil law system and common law system in five aspects: legal source, demonstration method, judicial power, evidence source and trial mode (23, 24). Due to the differences of legal systems, the impact of BITs on contract execution in different developing countries may be significantly different. For the 73 developing countries selected in this paper, 26 countries such as Albania and Argentina belong to the civil law system, while 47 countries such as Rwanda and South Africa belong to the common law system. In order to further test the differential impact of BITs on contract execution in developing countries of civil law system and common law system, this paper will conduct the regression based on different legal system. Table 3 reports specific regression results.

**Table 3:** Results of civil law system and common law system.

	**Civil Law System**		**Common Law System**	
	**(1)**	**(2)**	**(3)**	**(4)**
*ln treaties*	0.052^**^ (2.33)	0.039^**^ (2.01)	0.122^***^ (3.05)	0.108^***^ (2.69)
*ln pgdp*		−0.010 (-0.65)		−0.023 (-1.25)
*lnopen*		0.029^***^ (2.70)		0.039^*^ (1.90)
*resources*		0.056** (2.42)		0.059^***^ (2.65)
*control*		−0.008^**^ (-2.16)		−0.012^**^ (-2.37)
constant	2.790^***^ (62.32)	2.451 (19.56)	4.021^***^ (69.45)	3.838^***^ (18.16)
R^2^	0.2231	0.2145	0.2930	0.2882
Observations	390	390	705	705

***, **, * *means significant at 1, 5, and 10% levels respectively. The values reported in parentheses are t-values*.

From the results of columns (1) and (2), it can be seen that compared with developing countries of civil law system, BITs have a significant impact on contract execution in developing countries of common law system. First of all, the common law system emphasizes the freedom of contract more. The parties to the contract often have greater discretion over the specific arrangement of the contract, and the law rarely adds other mandatory contents to the contract. In most developing countries with civil law system, the legal system of the host country usually endows the contract with some “implied terms”. Taking contract execution as an example, although the contract does not contain specific contents and terms, both parties still need to take the bona fide execution of the contract as the basis (25). Secondly, in the developing countries of common law system, “civil contract” and “administrative contract” are governed by private law, and they are not treated differently. However, in many developing countries of civil law system, the concept of “administrative contract” has been introduced into their legal system, and special legal institutions have been set up to adjudicate disputes. Due to the differences in the freedom of contract and the construction of legal system between the developing countries of civil law system and common law system, the impact of multinational enterprises on the contract execution of the host country through BITs is significantly different.

## Robustness Tests

In order to ensure the robustness of the core conclusion, this paper will test it from the perspective of research methods and data. Firstly, although the fixed effect estimation can avoid the endogenous problem caused by missing variables to a certain extent, for the sake of robustness, this paper will choose the variable with two-stage lag of BITs as the instrumental variable, and retest the core conclusion by using two-stage least square method (2SLS) and generalized method of moments (GMM). According to the results in columns (1)–(4) of Table 4, the coefficients of BITs are positive and have passed the significance test at the level of 1%, indicating that the overall and effective parts of BITs can significantly improve the contract execution of developing countries, that is, the core conclusion of this paper is relatively stable. From the test results of instrumental variables, the Cragg-Donald F and Kleibergen-Paap F statistics of weak instrumental variables are greater than the critical value under the 10% error level, indicating that there is no weak instrumental variable. The P values of FAR test are >10%, which rejects the assumption that the instrumental variables selected in this paper are exogenous.

**Table 4:** Robustness test I.

	**BITs in General**		**BITs in Force**	
	**(1)**	**(2)**	**(3)**	**(4)**
	**IV-2SLS**	**IV-GMM**	**IV-2SLS**	**IV-GMM**
*ln treaties*	0.043^***^ (2.92)	0.043^***^ (2.92)	0.050^***^ (3.16)	0.050^***^ (3.16)
control variables	YES	YES	YES	YES
FAR Test	0.203	0.203	0.227	0.227
Cragg-Donald F Wald F Statistic	21.65 [8.08]	22.73 [8.08]	26.31 [8.13]	25.80 [8.13]
Kleibergen-Paap rk Wald F Statistic	15.77 [8.08]	15.77 [8.08]	18.26 [8.13]	18.26 [8.13]
constant	1.676^***^ (6.42)	1.676^***^ (6.42)	1.053^***^ (3.87)	1.053^***^ (3.87)
R^2^	0.3036	0.3036	0.2904	0.2904
Observations	1,022	1,022	1,022	1,022

*The values reported in brackets are critical values of Stock and Yogo (2005) under the 10% level.*

***, **, * *means significant at 1, 5, and 10% levels respectively. The values reported in parentheses are t-values*.

According to the United Nations, there are 36 developed countries in the world. In the research sample of this paper, there are seven countries such as South Korea, Poland and Kuwait, which belong to high-income countries, that is, the per capita GDP is more than 12,000 US dollars. Compared with other developing countries, the economic and social development of these seven countries is obviously better. Out of concern about the abnormal data, this paper excludes the above seven high-income countries and re-estimates the data. It can be seen from the estimation results in Table 5 that the overall BITs and the BITs that have entered into force have not only passed the significance test, but also their coefficients have improved. This result shows that the core conclusion of this paper is relatively robust. In addition, the increase of the coefficient of BITs shows that it has a greater improvement effect on the contract execution of developing countries with low economic development.

**Table 5:** Robustness test II.

	**BITs in General**		**BITs in Force**	
	**(1)**	**(2)**	**(3)**	**(4)**
*ln *treaties**	0.123^***^ (3.78)	0.092^**^ (2.52)	0.135^***^ (4.99)	0.101^***^ (2.80)
control variables	YES	YES	YES	YES
constant	3.019^***^ (45.07)	2.821^***^ (19.16)	3.131^***^ (52.00)	2.054^***^ (12.23)
R^2^	0.2885	0.3007	0.2902	0.3251
Observations	990	990	990	990

***, **, * *means significant at 1, 5, and 10% levels respectively. The values reported in parentheses are t-values*.

## Further Research

Based on the above research results, it can be found that both the total number of BITs and the effective part of them play a positive role in promoting the business environment of developing countries. Although this study examines the overall impact of BITs on the contract execution, it ignores their impact on various specific aspects, so there are certain deficiencies. Based on this, this paper will further analyse the impact of BITs on specific aspects of the contract execution, such as time and cost. Because there is no significant difference between the total quantity of BITs and the effective part, and the former covers a more comprehensive scope, this paper chooses the total quantity of BITs as the measurement indicator. In addition, the results of the Hausman test show that fixed-effects estimation should be used in the regression analysis of these nine specific aspects. Specific regression results are shown in Table 6.

**Table 6:** Results of time and cost.

	**ln ***time*****				**ln ***cost*****			
	**BITs in General**		**BITs in Force**		**BITs in General**		**BITs in Force**	
	**(1)**	**(2)**	**(1)**	**(2)**	**(3)**	**(4)**	**(3)**	**(4)**
*ln treaties*	0.142^***^ (5.86)	0.106^***^ (3.78)	0.120^***^ (5.50)	0.089^***^ (3.62)	0.086^**^ (2.18)	0.075^*^ (1.93)	0.099^***^ (2.99)	0.084^**^ (2.29)
*ln pgdp*		0.013 (1.24)		0.015 (1.40)		−0.017 (−1.26)		−0.022 (−1.61)
*ln trade*		0.036^**^ (2.11)		0.038^**^ (2.20)		0.069^***^ (3.14)		0.067^***^ (3.58)
*ln control*		0.086^***^ (2.69)		0.087^***^ (2.73)		0.128^***^ (3.12)		0.096^**^ (2.33)
*ln resources*		0.004 (0.62)		0.004 (0.72)		−0.022^***^ (−2.94)		−0.026^***^ (−3.41)
*ln urban*		0.055 (1.16)		0.054 (1.14)		0.406^***^ (5.84)		0.391^***^ (5.61)
constant	3.671^***^ (44.59)	2.982^***^ (14.12)	3.780^***^ (5.50)	3.040^***^ (14.28)	3.766^***^ (26.88)	1.819^***^ (5.89)	3.755^***^ (33.27)	3.071^***^ (14.35)
R^2^	0.3405	0.3804	0.3012	0.3561	0.3274	0.3519	0.3061	0.3138
Observations	1,095	1,095	1,095	1,095	1,095	1,095	1,095	1,095

***, **, * *means significant at 1, 5, and 10% levels respectively. The values reported in parentheses are t-values*.

In terms of time, the overall coefficients of BITs and effective BITs are significantly positive, which shows that both play a significant role in promoting. Compared with BITs as a whole, the promotion effect of BITs that have entered into force is relatively stronger. Compared with BITs in general, the direct impact effect of effective BITs is stronger, which shows that the specific aspect of BITs for time is mainly through direct impact. In terms of cost, the overall coefficients of BITs and effective BITs are also positive, and have passed the significance test at least at the 10% level, that is, both can significantly promote the improvement of the cost of host countries. Different from the time, the overall impact of BITs on the cost is slightly stronger than that of the effective BITs, indicating that the impact of BITs on the cost may be mainly through reference. It should be noted that the impact of BITs on cost is significantly stronger than time, indicating that capital exporting countries pay more attention to cost rather than time spent in contract execution.

## Conclusion

BITs are an important legal guarantee for capital exporting countries to safeguard their own economic interests in the international economic and trade system. Contract execution is not only an important part of the business environment of developing countries, but also directly related to the actual operating benefits of multinational enterprises and local enterprises. Taking the data of 73 developing countries from the world bank's business environment project and the United Nations Conference on Trade and Development from 2005 to 2019 as research samples, this paper discusses the improvement effect of BITs on contract execution in developing countries. The empirical study found that the BITs in general or the BITs that have entered into force can play a positive role in promoting the contract execution of developing countries. Compared with the developing countries of civil law system, the developing countries of common law system have greater freedom of contract and less potential legal constraints. Therefore, whether the BITs are in force or in general, the improvement effect of BITs on the contract execution of developing countries belonging to common law system is more prominent. After the 2008 financial crisis, the global macro-economy fell into turmoil. In order to protect their rights and interests to a greater extent, multinational enterprises reflect a more significant institutional dependence, that is, the overall BITs and the effective BITs reflect a more significant improvement effect of contract execution in the post financial crisis period. In terms of the impact of BITs on the specific aspects of contract execution in developing countries, BITs have a significant impact on both time and cost, but the improvement effect on time is more obvious.

This issue can be important in the current COVID-19 crisis, and we should expect that BITs will improve the quality of the contract execution in developing countries in the post-COVID-19 era.

## Data Availability Statement

The original contributions presented in the study are included in the article/supplementary material, further inquiries can be directed to the corresponding author.

## Author Contributions

ZY is responsible for the idea and organization of this paper. CZ is responsible for empirical part. KS is responsible for the theoretical analysis. MZ and QW are responsible for the data collection. All authors contributed to the article and approved the submitted version.

## Conflict of Interest

The authors declare that the research was conducted in the absence of any commercial or financial relationships that could be construed as a potential conflict of interest.

## Publisher's Note

All claims expressed in this article are solely those of the authors and do not necessarily represent those of their affiliated organizations, or those of the publisher, the editors and the reviewers. Any product that may be evaluated in this article, or claim that may be made by its manufacturer, is not guaranteed or endorsed by the publisher.
